# Frequency of conjunctival epithelial dysplasia in patients with pterygium

**DOI:** 10.5935/0004-2749.20200053

**Published:** 2020

**Authors:** Daniel Lomelí-Linares, Lissete García-Salgado, Gabriela Riancho-Sánchez, Ellery Lopez-Star, Van C. Lansingh, Sonia Corredor-Casas

**Affiliations:** 1 Oculoplastic and General Ophthalmology Department, Instituto Mexicano de Oftalmologia IAP Queretaro, Queretaro, Mexico; 2 Regional Hospital Adolfo Lopez Mateos ISSSTE, Mexico City, Mexico; 3 Private practice, Queretaro, Queretaro, Mexico; 4 Instituto Mexicano de Oftalmología IAP, Queretaro, Querétaro, México; 5 Ophthalmic Pathology Department, Instituto Mexicano de Oftalmología IAP, Querétaro, Mexico

**Keywords:** Pterygium, Conjunctival neoplasms, Eye neoplasms, Histopathology, Carcinoma, squamous cell, Pterígio, Neoplasias da túnica conjuntiva, Neoplasias oculares, Histopatologia, Carcinoma de células escamosas

## Abstract

**Purpose:**

To determine the frequency of ocular squamous surface neoplasia associated with pterygium in an ophthalmology reference center in Central Mexico.

**Methods:**

We reviewed histopathological reports and slides of all patients who underwent pterygium surgery from 2014 to 2016 at the Instituto Mexicano de Oftalmologia in Queretaro (Mexico).

**Results:**

We studied 177 biopsy samples; 66% were from women, and the median age was 52 years. We found ocular squamous surface neoplasias in 11.29% (n=20) of the samples. One biopsy sample revealed a poorly differentiated keratinizing and infiltrating carcinoma.

**Conclusions:**

The prevalence of ocular squamous surface neoplasia in our region appears to be high. Countrywide studies are necessary to determine the true prevalence of ocular squamous surface neoplasia in Mexico and to examine related risk factors.

## Abstract (pt)

**Objetivo:**

Determinar a frequência de neoplasia escamosa da superfície ocular associada ao pterígio com apresentação clínica, em um centro de referência em Oftalmologia da região central do México.

**Métodos:**

Revisamos os laudos histopatológicos e as lâminas de biópsia de todos os pacientes que foram submetidos à cirurgia de pterígio de 2014 a 2016 no Instituto Mexicano de Oftalmologia, na cidade de Querétaro.

**Resultados:**

Estudamos 177 amostras de biópsia; 66% eram de pacientes do sexo feminino, sendo a mediana da idade de 52 anos. Encontramos neoplasia escamosa da superfície ocular em 11,29% (n=20). Uma amostra de biópsia mostrou um carcinoma queratinizante infiltrativo pouco diferenciado.

**Conclusões:**

A prevalência da neoplasia escamosa da superfície ocular nessa região parece ser maior do que a indicada por outras pesquisas. Mais estudos de âmbito nacional são necessários para determinar a verdadeira prevalência da neoplasia escamosa da superfície ocular no México e examinar os fatores de risco relacionados.

## INTRODUCTION

Pterygium is a fibrovascular proliferation originating in the conjunctival substantia propria. It grows in a horizontal pattern from the bulbar conjunctiva toward the cornea. In advanced cases, it may alter vision and cause red eye and conjunctival irritation as a result of inflammation. Pterygiums can also transform into cancers such as an ocular surface squamous neoplasia (OSSN)^(1)^.

Locations with high ultraviolet (UV) light exposures present high incidences of pterygium, as in the equatorial belt covering the latitudinal area from 37**°** north to 37**°** south^(2)^. Approximately 22% of the individuals living in equatorial zones have pterygiums, compared with only 2% of the individuals outside of this geographical zone (at higher latitudes). This statistic supports the hypothesis that UV light plays an important role in the genesis of pterygiums^(3)^. A higher prevalence of pterygium is also reported among rural populations than among urban residents, probably due to higher occupational sun exposure in the first group of individuals. Likewise, individuals older than 60 years have the highest inci dence of pterygium (up to 20%), and this may be explained by the cumulative effect of unprotected sun exposure on the tissues^(4)^.

The Chesapeake Bay Study found a significantly higher risk of developing pterygium in individuals with higher UV-A and UV-B exposures than in others^(5)^. UV exposure enhances the formation of pyrimidine dimers, thus affecting the DNA repair process and favoring the development of OSSN, which includes dysplasia, in situ carcinoma, and invasive conjunctival or epidermoid carcinoma.

OSSN incidence varies from 0.13 to 0.19 per 100,000 population, depending on the geographical zone, so timely diagnosis and treatment are extremely important to avoid progression and complications such as orbital invasion and metastases^(6)^.

OSSNs may be misdiagnosed in patients with pterygium; thus, we aimed to determine the frequency of these lesions in patients diagnosed as having a pterygium but without signs of OSSN^(7)^ and to assess age, gender, and other demographic characteristics of the individuals in our sample to identify any significant correlations with the presence of OSSN.

## METHODS

We conducted a retrospective, transversal, and observational study with the approval of the Research Ethics Committee. We enrolled patients with a clinical pterygium diagnosis, but lacking clinical suspicion of conjunctival neoplasia, who underwent pterygium resections between 2014 and 2016.

**Figure 1:**
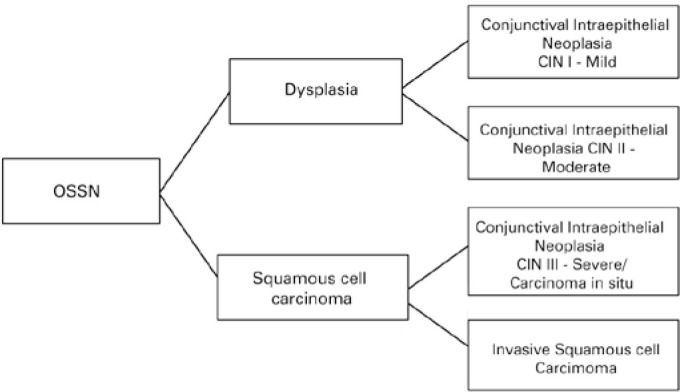
Classification of ocular surface squamous neoplasia (OSSN) used in this studyFrom mild dysplasia to carcinoma in situ and squamous cell carcinoma.

We used the following histopathological criteria to classify the OSSN lesions (Figure 1): Conjunctival intraepithelial neoplasia (CIN) grade I (mild dysplasia circumscribed to the basal third of the conjunctival-corneal epithelium), CIN grade II (moderate dysplasia restricted to the basal two-thirds of the conjunctival-corneal epithelium), CIN grade III/SCC in situ (severe dysplasia embracing the whole thickness of the conjunctival-corneal epithelium)^(6)^.

A single observer performed the histopathological evaluations using the diagnostic criteria mentioned. We would like to emphasize that this observer is one of only eight certified pathologists currently active in Mexico, so she is likely the most qualified person in our country to examine this kind of biopsy and few other professionals possess a similar level of knowledge and experience analyzing these types of samples.

### Inclusion criteria

- Patients with a pterygium diagnosis scheduled for resection between 2014 and 2016 at the Instituto Me xicano de Oftalmología IAP.

### Exclusion and elimination criteria

- Patients with a pterygium diagnosis who declined the resection.
- Patients with recurrent pterygiums.
- Biopsy collection and analysis outside of the established time period.
- Patients with conjunctival or ocular surface pathology, previous surgery, or trauma.
- Patients with incomplete files.
- Insufficient pterygium sample.
- Inconclusive biopsy results.

We collected data on age, gender, occupation, affected eye, pterygium location, severity grading, classification as primary or recurrent, clinical diagnosis, and histopathological diagnosis and features of the patients, and we analyzed them to find statistical correlations between the variables.

We recorded data using Microsoft Excel 2010^TM^ and used the SPSS^TM^ version 15.0 for Windows^TM^ to analyze descriptive population variables such as gender, age, and occupation and assessed correlations of those with the presence of OSSN using the Pearson Test.

## RESULTS

**Figure 2:**
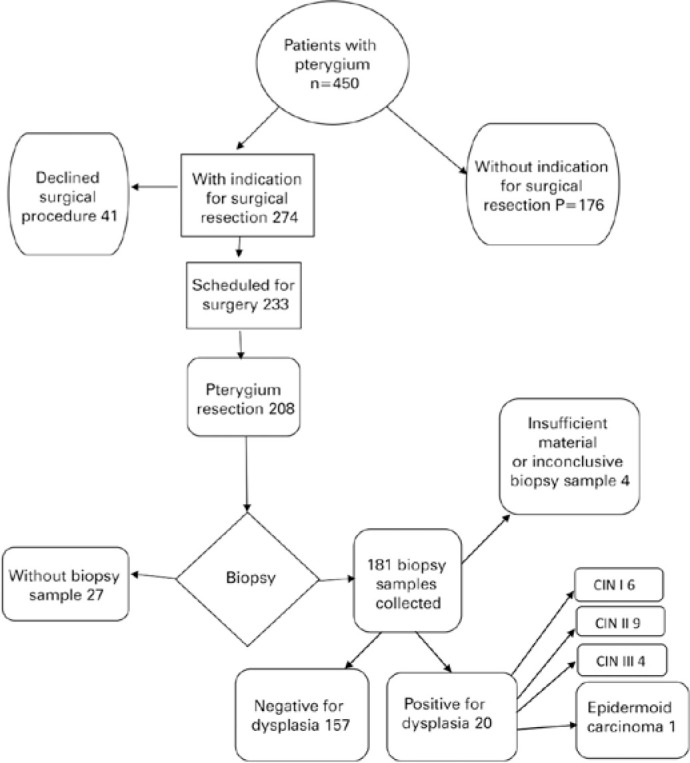
Flow chart of clinical study.

**Figure 3:**
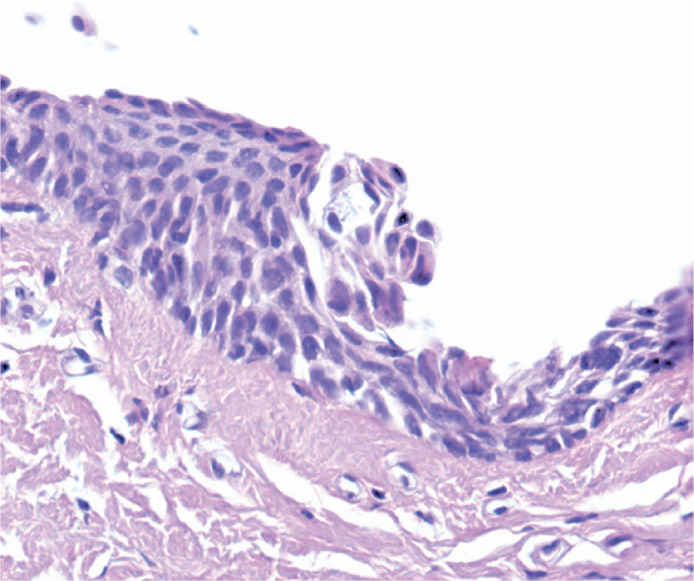
Photomicrograph showing a CIN Grade I lesion (mild dysplasia) H&E (40X).

**Figure 4:**
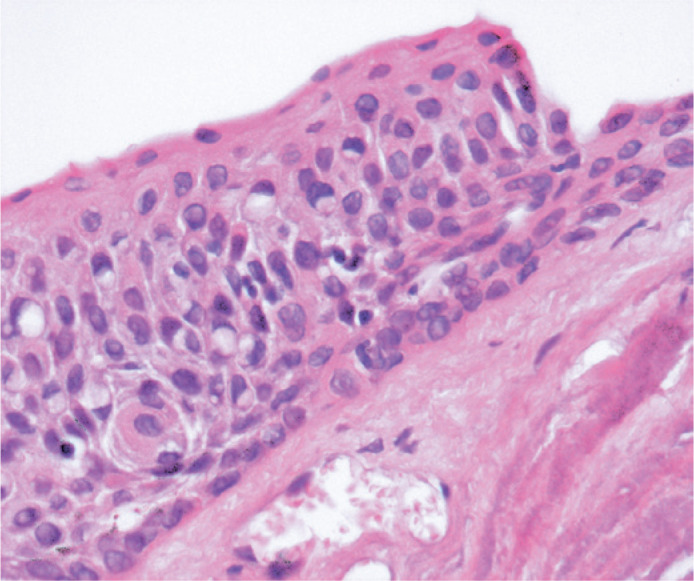
Photomicrograph showing a CIN Grade II (moderate dysplasia) affecting 2/3 of the basal conjunctival epithelium. H&E (40X).

**Figure 5:**
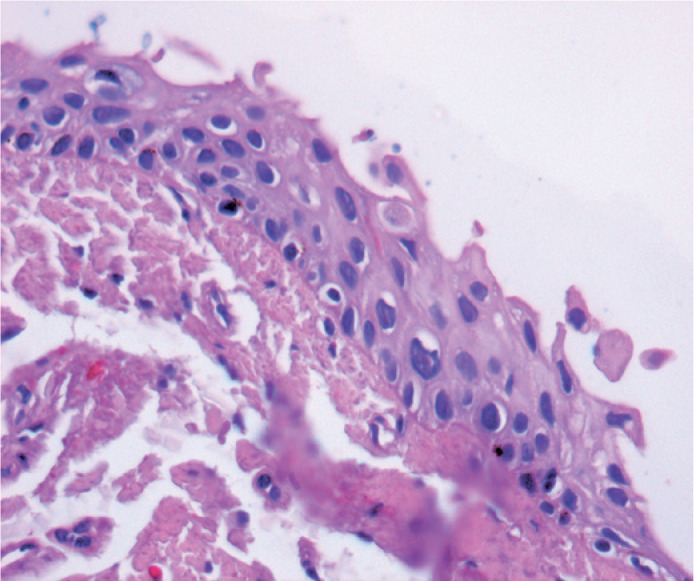
Histological cut showing dysplastic changes across the entire thickness of the conjunctival epithelium (CIN grade III/*in situ* carcinoma) H&E (40X).

**Figure 6:**
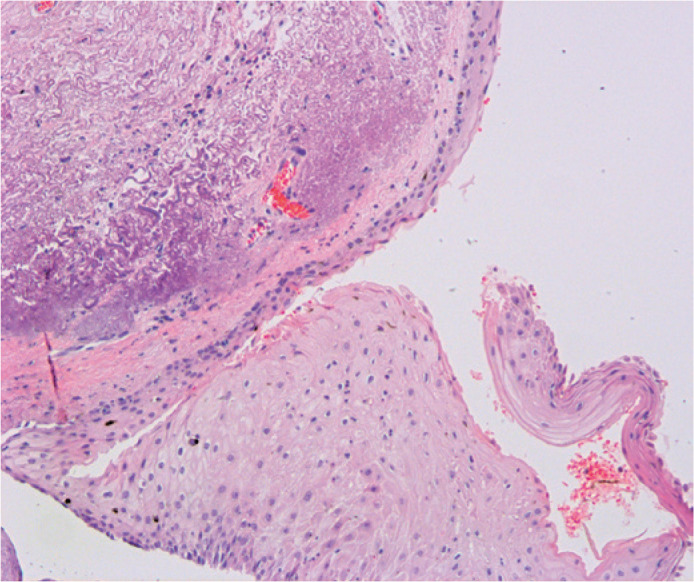
Microscopic appearance of a well-differentiated invasive squamous cell carcinoma

We collected 177 samples from 172 patients (Figure 2) and confirmed clinical diagnoses of pterygium in all of them. Most samples (66.25%; n=114) were from women, and 35.46% (n=61) were from men. Their average age was 52.06 years (ranging from 25 to 77 years). We found 88.71% (n=157) of the samples had pterygium diagnoses by histopathology, whereas 11.29% (n=20) had histopathological diagnoses of OSSN (Table 1), and from this group, 19 of them (10.73%) with some degree of CIN. The histological reports included six (3.38%) biopsy samples with CIN I (Figure 3), nine (5.08%) with CIN II (Figure 4), and four (2.25%) with CIN III (Figure 5). One biopsy sample (0.56%) showed features of a poorly differentiated (Figure 6) keratinizing and infiltrating epidermoid carcinoma. We found no significant correlation between the presence of OSSN and any of the variables tested.

**Table 1:** Frequency of conjunctival epithelial dysplasia in patients with pterygium

Total number of Samples	Laterality RE/LE	Location nasal/ temporal	OSSN
N=177	Right eye (n=72) 40.6%	Nasal (n=69) 38.98%	(n=11) 6.21%
		Temporal (n=3) 1.69%	(n=1) 0.56%
	Left eye (n = 105) 59.3%	Nasal (n=105) 59.32%	(n=8) 4.51%
		Temporal (n=0) 0%	(n=0) 0%

OSSN= ocular surface squamous neoplasia.

Transition zone between pterygium and neoplasia. H&E (5X).

## DISCUSSION

Our results show that the frequency of unsuspected OSSN in patients with pterygiums at the Instituto Mexicano de Oftalmología IAP located in Mexico’s Bajio region was 11.29% (n=9). This frequency is higher than frequencies reported in Thailand (1.8%)^(7)^, the U.S. (1.7%)^(8)^, Peru (0.65%)^(9)^, Australia (Sydney, 0%)^(10)^, and Canada (0%)^(11)^ but very similar to those reported in Australia (Queensland, 9.8%)^(1)^ and India^(12)^. However, we could not demonstrate correlations between specific occupations and the presence of OSSN, probably because the collected data related to UV occupational exposure were incomplete.

Areas with high exposure to UV light like Mexico located in the torrid zone have higher OSSN prevalences than areas with lower UV exposures. Thus, the prevalence in Mexico should be similar to those in other countries with similar tropical latitudes such as China, Singapore, Australia, and India. However, the Capiz Correa report, conducted among a Mexican population at the Nuestra Señora de la Luz Foundation Hospital, found a higher OSSN prevalence among patients older than 70 years and a higher prevalence in women (67.44%)^(13)^. The differences with our results may be explained by their analysis having been based on epidermoid carcinoma histopathological studies, whereas ours was based on patients with a pterygium diagnosis but without clinical suspicion of OSSN. We also found more patients with nasal OSSN than with temporal OSSN, probably because sunlight travels through the eye and is focused on the opposite corneal side near the nasal limbus.

Our results are similar to those reported by Hirst et al. (Australia 2009)^(1)^, who found an OSSN frequency of 9.8% (52 out of 533 individuals), with 63.5% (n=33) of cases having a mild dysplasia, 19.2% (n=10) a moderate dysplasia, 9.6% (n=5) a severe dysplasia, and 1.9% (n=1) an infiltrative epidermoid carcinoma.

The elevated OSSN frequency in our study (in patients without clinical suspicion) suggests the importance of histopathological examinations for all patients undergoing pterygium operations. Histopathological evaluations provide advantages: the most important one is the microscopic diagnosis (the gold standard) that allows for accurate therapeutic decisions (such as expanding surgical borders). They also provide feedback for continuing medical education, especially for residents who need to alert patients of the warning signs that may signal recurrences or the appearance of new lesions in high-exposure areas. In developed countries, surgically removed tissues undergo routine histopathological studies^(14)^.

The prevailing hypothesis is that a higher prevalence of OSSN exists in areas with high UV exposure. UV light from the sun is divided into UVA, UVB, and UVC^(15)^.

At 320-400 nm, UVA has the longest wavelength and higher penetration ability, and it is not attenuated by the ozone layer. UVA light represents 90%-99% of the UV radiation. Among its consequences are pigmentation, premature aging, immunosuppression, and carcinogenesis. Its biological effects in pterygiums include DNA damage, oxidative stress, and activation of independent cellular surface receptors^(15,16)^.

The initial pterygium phase is due to damage to limbus stem cells. First, light striking the temporal limbus focuses on the nasal limbus causing damage to limbus stem cells and fibroblasts in the nasal zone. Then, a centripetal migration of fibroblasts produces a migratory limbus. The development of a pterygium involves growth factors such as heparin-binding epidermal growth factor (promoting angiogenesis and stimulating anchorage growth and cellular transformation), vascular endothelial growth factor (increasing vascular permeability, angiogenesis, and lymphangiogenesis), platelet-derived growth factor (promoting fibroblast proliferation), and trans forming growth factor beta (promoting angiogenesis and fibroblast activation with collagen production)^(15,16)^.

P53 mutations are implicated in the neoplastic transformation of some human cells. Some researchers have reported a higher immunohistochemical expression of p53 in pterygium biopsies. This supports the current view that pterygiums should be considered neoplasias rather than a type of degenerative tissue^(3,16)^.

Risk factors for developing OSSN include smoking, human immunodeficiency virus (HIV) infection, human papillomavirus (HPV) type 16 or 18 infection, and UV light exposure^(17)^. The HPV virus inactivates the p53 gene. Different HPV serotype infections have been rep orted in some regions with a greater frequency of pterygium, but wide variation is shown among geographical areas and populations. HPV infection may play a role in pterygium genesis, but only in populations with a higher prevalence of the infection^(18)^. Although we found no associations in our Mexican population to factors such as HIV and HPV infections, many risk factors could be associated with OSSN^(19)^.

In a report from hospitals in Kenya between 2012 and 2014 (supported by the Moorfields Eye Hospital), the use of a vital staining technique (0.05% toluidine blue) is proposed as a clinical marker for the limits of dysplastic epithelial lesions of the conjunctiva with a level of 92% sensitivity for dysplasia detection^(20)^. This technique will be the subject of a more detailed study in the near future in our hospital.

Histopathological studies for all patients undergoing pterygium removal are important because the incidence of OSSN in patients without clinical suspicion is high.

Detailed photographic analyses of OSSN-diagnosed patients may be useful to discover clinical patterns indicating dysplasia, but cellular analyses using special stains such as toluidine blue are required to increase the sensitivity for detection. Optic coherence tomography of the conjunctiva may also help identify changes that suggest the beginning of OSSN^(21)^.

One of the deficiencies of our study is that some of the data entries for our patient files were incompletely or incorrectly collected. This makes it difficult to establish cause-effect associations to uncover the OSSN pathogenesis. Thus, we have now implemented new ways of collecting information from patients who have a pterygium diagnosis. For example, each patient will be asked about his or her occupation, sun exposure in hours per week, and the use of sunscreen, sunglasses, a hat, or other types of eye protection.

The prevalence of OSSN in patients with pterygium but without clinical suspicion of OSSN is high. Histopathological studies of excised pterygium tissues often show the presence of conjunctival intraepithelial lesions considered to be precancerous, or epidermoid carcinomas, and these are usually underdiagnosed based on clinical criteria alone. In other words, many patients with pterygium may present OSSNs (up to 11.29% of them).

Training medical staff to identify early clinical signs of dysplasia in pre-existent conjunctival lesions may lead to early diagnoses.

Broader studies in the general population are necessary in zones geographically similar to Mexico’s Bajio region, in order to assess the true prevalence of OSSN in Mexico.
